# Single-step genome-wide association study for social genetic effects and direct genetic effects on growth in Landrace pigs

**DOI:** 10.1038/s41598-020-71647-x

**Published:** 2020-09-11

**Authors:** Joon-Ki Hong, Jae-Bong Lee, Yuliaxis Ramayo-Caldas, Si-Dong Kim, Eun-Seok Cho, Young-Sin Kim, Kyu-Ho Cho, Deuk-Hwan Lee, Hee-Bok Park

**Affiliations:** 1 National Institute of Animal Science, Rural Development Administration, Cheonan, 31000 Republic of Korea; 2 Korea Zoonosis Research Institute, Chonbuk National University, 54531 Iksan, Republic of Korea; 3 Animal Breeding and Genetics Program, Institute for Research and Technology in Food and Agriculture (IRTA), Torre Marimon, 08140 Caldes de Montbui, Spain; 4 Department of Animal Life Resources, Hankyong National University, Anseong, 17579 Republic of Korea; 5 Department of Animal Resources Science, Kongju National University, Yesan, 32439 Republic of Korea

**Keywords:** Genetics, Agricultural genetics, Animal breeding, Genetic association study, Genetic markers, Genomics, Heritable quantitative trait, Systems biology, Biochemical networks

## Abstract

In livestock social interactions, social genetic effects (SGE) represent associations between phenotype of one individual and genotype of another. Such associations occur when the trait of interest is affected by transmissible phenotypes of social partners. The aim of this study was to estimate SGE and direct genetic effects (DGE, genetic effects of an individual on its own phenotype) on average daily gain (ADG) in Landrace pigs, and to conduct single-step genome-wide association study using SGE and DGE as dependent variables to identify quantitative trait loci (QTLs) and their positional candidate genes. A total of 1,041 Landrace pigs were genotyped using the Porcine SNP 60K BeadChip. Estimates of the two effects were obtained using an extended animal model. The SGE contributed 16% of the total heritable variation of ADG. The total heritability estimated by the extended animal model including both SGE and DGE was 0.52. The single-step genome-wide association study identified a total of 23 QTL windows for the SGE on ADG distributed across three chromosomes (i.e., SSC1, SSC2, and SSC6). Positional candidate genes within these QTL regions included *PRDM13*, *MAP3K7*, *CNR1*, *HTR1E*, *IL4*, *IL5*, *IL13*, *KIF3A*, *EFHD2*, *SLC38A7*, *mTOR*, *CNOT1*, *PLCB2*, *GABRR1*, and *GABRR2*, which have biological roles in neuropsychiatric processes. The results of biological pathway and gene network analyses also support the association of the neuropsychiatric processes with SGE on ADG in pigs. Additionally, a total of 11 QTL windows for DGE on ADG in SSC2, 3, 6, 9, 10, 12, 14, 16, and 17 were detected with positional candidate genes such as *ARL15.* We found a putative pleotropic QTL for both SGE and DGE on ADG on SSC6. Our results in this study provide important insights that can help facilitate a better understanding of the molecular basis of SGE for socially affected traits.

## Introduction

Livestock are usually raised in groups. Accordingly, social interactions and/or hierarchy of animals in the group can affect phenotypic variations such as growth rate. Competition is a ubiquitous example of social interaction and can cause considerable negative impacts on productivity (e.g., decreased feed efficiency and reduced weight gain) and animal welfare^1^. Although explicit observation and evaluation of the impact of social interactions on a phenotype is challenging, a quantitative genetic model was proposed that can decompose phenotypic values into a direct effects that originated from an individual’s own characteristics and indirect effects (i.e., social effect) that are derived from others^2^. As the direct effect can be partitioned into an inheritable component (i.e., direct genetic effect, DGE) and an environmental component, the indirect effect also can be partitioned into a genetic component and an environmental component. The genetic components of an indirect effect are called indirect genetic effects, or social genetic effects (SGE). Hence, SGE represent associations between phenotype of one individual and genotype of another.

The SGE can be estimated using linear mixed model frameworks by incorporating SGE as a random effect together with the conventional random DGE^3^. In this extended best linear unbiased prediction (BLUP) model, the random SGE can be considered as competitive effects. In 2002, Muir and Schinckel showed that the incorporation of the competitive effects into their extended BLUP model used for Japanese quail breeding resulted in substantially increased selection response compared with the conventional BLUP approach that incorporated only DGE^4^. Subsequently, more comprehensive linear mixed models were developed to better estimate both DGE and SGE^5–7^. SGE have been estimated and shown to be responsible for many phenotypes across several animal species, including pigs^8^, chickens^9^, quail^5^, deer^10^, mink^11^, and laboratory mice^12^. In particular, the magnitude of SGE in pigs has been estimated for average daily gain (ADG)^8,13,14^, feed intake and muscle depth^8^, and carcass traits^15^; these studies reported that the genetic variation in pen mates (i.e., SGE) substantially influenced the phenotypic variation of diverse traits in these animals.

Thanks to the noteworthy advancements in molecular and statistical genetic methodologies, genome-wide association studies (GWASs) to detect quantitative trait loci (QTLs) and their positional candidate genes have become feasible^16,17^. Until recently, however, a limited number of GWASs have been performed to identify QTLs responsible for SGE, and little is known about their genetic architecture or individual genes implicated^18,19^. The aims of this study were to estimate variance components of SGE and DGE for ADG in purebred Landrace pigs and to perform a single-step GWAS (ssGWAS) to identify QTLs and positional candidate genes associated with the traits of interest. In addition, post-GWAS functional annotation analyses (i.e., pathway and network-based analyses) were conducted to elucidate the biological background of the underlying SGE using the list of positional candidate genes identified based on the ssGWAS results.

## Results and discussion

### Variance components and genetic parameters of SGE and DGE

The (co)variances and parameters obtained from the studied model for Landrace pigs are presented in Table 1. The total heritability ($T^{2}$) estimated by the extended BLUP model including both DGE and SGE was 0.52 in our Landrace population. This value was greater than classical heritability ($h^{2}$) in this pig breed (0.36). Moreover, the SGE, $\left( {n{ }{-}{ }1} \right)^{2} \sigma_{{a_{S} }}^{2}$, contributed 16% of the total heritable variation ($\sigma_{TBV}^{2}$) of ADG, which is available for response to selection in Landrace pigs. The correlation coefficient between DGE and SGE was moderate ($r_{{a_{DS} }}$ = 0.23). The $h^{2}$ and $T^{2}$ estimates in our study were higher than those of the previous studies^7,8^. An explanation of the observed differences is due to the fact that the breeder of these maternal lines for pig selection have focused on reproductive traits, such as litter size, there has been little chance of genetic improvement for growth. Bergsma et al. suggested that the absence of competition between mate growth and an individual’s own growth might be a result of neutral or marginally cooperative social interactions^8^. According to Bijma et al., the positive covariance between direct variance and indirect (i.e., social genetic) variance is likely to increase the $\sigma_{TBV}^{2}$, which well corresponds to the findings of this study^6^. Canario et al*.* reported that individuals may improve the growth of their social mates without personal cost^7^. In this study, the positive correlation in Landrace pigs could suggest that their social partners might have stimulated a greater ADG. Moreover, in addition to being influenced by environmental factors, this results indicate that SGE contribute to the additive genetic component together with DGE. Therefore, the GWAS was conducted to delineate the genetic architecture of SGE and DGE in Landrace pigs.

**Table 1:** Estimates of variance components and genetic parameters for average daily gain in Landrace pigs.

(Co)variance components and genetic parameters	Estimate (posterior standard deviation)
$\sigma_{{a_{D} }}^{2}$^a^	2,235 (168)
$\sigma_{{a_{S} }}^{2}$	16 (4)
$\sigma_{p}^{2}$	6,260 (97)
$\sigma_{TBV}^{2}$	3,261 (402)
$h^{2}$	0.36 (0.02)
$T^{2}$	0.52 (0.06)
$r_{{a_{DS} }}$	0.23 (0.13)
$r_{lk}$	0.61 (0.17)
$\sigma_{c}^{2}$	43 (13)
$\sigma_{g}^{2}$	227 (51)
$\sigma_{l}^{2}$	218 (38)
$\sigma_{k}^{2}$	17 (5)
$\sigma_{pe}^{2}$	48 (27)
$\sigma_{e}^{2}$	3,299 (95)
$\sigma_{{a_{DS} }}$	43 (24)
$\sigma_{lk}$	36 (9)

^a^$\sigma_{{a_{D} }}^{2}$, direct genetic variance; $\sigma_{{a_{S} }}^{2}$, social genetic variance; $\sigma_{p}^{2}$, phenotypic variance; $\sigma_{TBV}^{2}$, total heritable variance; $h^{2}$ = $\sigma_{{a_{D} }}^{2}$ / $\sigma_{P}^{2}$, classical heritability; $T^{2}$ = $\sigma_{TBV}^{2}$ / $\sigma_{P}^{2}$, total heritability for the extended BLUP model including SGE; $r_{{a_{DS} }}$, correlation between direct and social genetic effects; $r_{lk}$, correlation between birth litter and early-life environmental effects; $\sigma_{c}^{2}$, random pen variance; $\sigma_{g}^{2}$, random group variance; $\sigma_{l}^{2}$, random litter variance; $\sigma_{k}^{2}$, random early-life environmental variance; $\sigma_{pe}^{2}$, random permanent variance; $\sigma_{e}^{2}$, residual variance; $\sigma_{{a_{DS} }}$, covariance between direct and social genetic effects; $\sigma_{lk}$, covariance between birth litter and early-life environmental effects.

### GWAS of SGE and DGE on ADG

#### SGE on ADG

**Figure 1:**
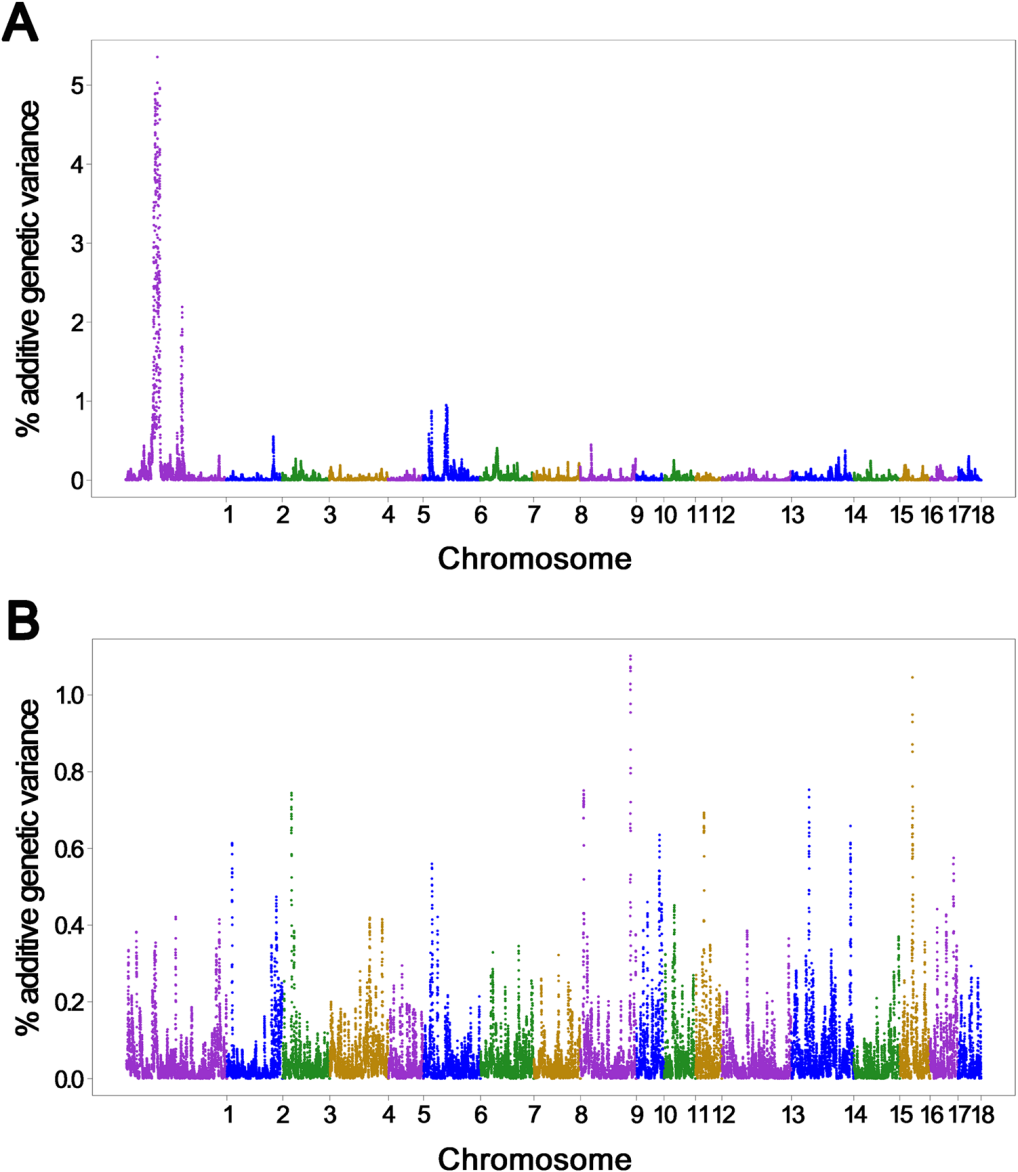
Results of the single-step GWAS (ssGWAS) for SGE and DGE on ADG in Landrace pigs. The X-axis shows the chromosomes, and Y-axis represents the proportion of additive genetic variance explained by 1.0 Mb window of adjacent SNPs for the phenotypes of interest. (**A**) GWAS plot of the ssGWAS for SGE on ADG (**B**) GWAS plot of the ssGWAS for DGE on ADG.

Non-overlapping 1-Mb windows that explained more than 0.5% of the additive genetic variance were determined to be QTLs for SGE on ADG in this study. The Manhattan plot of the single-step GWAS (ssGWAS) for SGE is shown in Fig. 1A. Following this criterion, we identified a total of 23 QTL windows for SGE on ADG distributed across three chromosomes (i.e., SSC1, SSC2, and SSC6) (Supplementary Table S1).

The top QTL, which explained 5.36% of the additive genetic variance, was mapped on SSC1 at the 61–62 Mb region. However, this window did not harbour any known gene. Among the 22 other QTLs, sixteen were also mapped on SSC1. The QTL window on SSC1 at 66–67 Mb explained the 2nd highest percentage of additive genetic variance (4.96%) for SGE on ADG and included a positional candidate gene, *PRDM13*. It has been known that GABA (gamma-aminobutyric acid) plays a critical role in the control of neurotransmitters as an inhibitor of neuronal activities related with neuropsychiatric processes^20^. *PRDM13* is required to generate the precise number of GABA (gamma-aminobutyric acid) associated neurons (i.e., GABAegic neurons)^21^. There is a report that a microdeletion of *PRDM13* locus has been implicated in autism and intellectual disability in human^22^. The QTL window on SSC1 at 58–59 Mb includes *MAP3K7*, which is known to be involved in autophagy. Autophagy is known to be related to psychiatric diseases such as schizophrenia^23^. *CNR1*, which encodes the type 1 cannabinoid receptor protein, is localised to the QTL window on SSC1 at 55.6–56.6 Mb. This window accounts for 3.52% of additive genetic variance for SGE on ADG. The endocannabinoid system plays a crucial role in the regulation of neurological activity throughout the central nervous system^24^. This gene is known to be associated with neurological phenotypes in mice^25^ and humans^26^. Also on SSC1, another QTL window at 56.9–57.9 Mb, accounting for 1.21% of additive genetic variance, harboured *GABRR1* and *GABRR2*, which encode receptors for GABA. The GABA makes use of its effects via their receptors. Hence, GABA receptors influence neurological and behavioural phenotypes in many animal species^27–29^. It was reported that *GABRR2* is a plausible positional candidate gene on SGE of feed conversion rate in Yorkshire pigs^19^. The QTL window on SSC1 at 54.4–55.4 Mb, accounting for 1.09% of additive genetic variance, harboured *HTR1E*, which encodes 5-hydroxytryptamine 1E receptor protein. The 5-hydroxytryptamine, which is also known as serotonin, plays an important role in mood control^30^, *HTR1E* has been implicated in autism spectrum disorders^31^. On SSC1, the QTL window at 51.5–52.5 Mb, explaining 0.58% of additive genetic variance, contained *RIMS1*. The protein encoded by *RIMS1* is a RAS gene superfamily member that controls synaptic vesicle exocytosis^32^ and influences behavioural traits linked to schizophrenia^33^.

On SSC2, only one QTL at the 134–135 Mb region was identified. This QTL region accounted for 0.55% of additive genetic variance for SGE on ADG. Three genes are located in this region that encode IL4, IL5, and IL13. These interleukins are involved in T cell receptor signaling pathway^34^. It is well documented that T cells are linked to the homeostasis of the central nervous system and therefore may be related to neurological or psychological traits^35,36^. *KIF3A* was also located in this region; this gene encodes the kinesin-like protein 3A, which has been implicated in a disorder involving motor neuron degeneration^37^. Furthermore, this gene was identified as a component of gene networks associated with conditional fear phenotypes in mice^38^.

On SSC6, five windows were identified as QTLs. The most significant QTL on SSC6 was located in the 73.7–74.7 Mb region, and explained 0.95% of additive genetic variance for SGE on ADG. *EFHD2*, which encodes EF hand domain containing 2 protein, is located in this QTL. *EFHD2* is expressed in human and mice brains. EFHD2 protein is a Ca^2+^ sensor protein which is likely involved in synaptic plasticity and affects various behavioural traits in mice^39^. Another QTL window at 19.9–20.9 Mb, which accounted for 0.87% of additive genetic variance, contained *SLC38A7*, which encodes solute carrier family 38 member 7 (also known as the SNAT7 protein). Hägglund et al. reported that SLC38A7 is a sodium-coupled amino acid transporter in glutamatergic neurons in the brain^40^, and it has been implicated as a risk-conferring glutamatergic gene in schizophrenia^41^. In the QTL region of SSC6:70.8–71.8 Mb, the mammalian target of rapamycin (mTOR) gene was found to be associated with SGE on ADG. mTOR is a broadly expressed serine-threonine kinase that coordinates major cellular processes, including synaptic and autophagy activation. Altered mTOR signal transduction is known to be associated with neuropsychiatric disorders such as schizophrenia^42^.

#### DGE on ADG

Eleven QTL windows that affected DGE on ADG were identified by ssGWAS. These QTLs were located on SSC2, 3, 6, 9, 10, 12, 14, 16, and 17 (Fig. 1B, Supplementary Table S2). Among the identified QTLs, the top QTL on SSC9 (128.2–129.2 Mb) accounted for 1.10% of the additive genetic variance of DGE on ADG. No obvious positional candidate gene for growth-related traits was found in this QTL region. The QTL that explained the 2nd highest percentage of additive genetic variance (1.05%) for DGE on ADG was located at 33–34 Mb of the SSC16, and included a positional candidate gene, *ARL15* encoding a small GTP-binding protein. In humans, the *ARL15* locus was found to be associated with plasma insulin, HDL cholesterol, adiponectin levels, and obesity. Moreover, a functional characterisation revealed that ARL15 influences adiponectin secretion and adipocyte differentiation^43^.

To evaluate the presence of putative pleiotropic QTL regions associated with SGE and DGE on ADG, we compared the two GWAS results; only one QTL (i.e., SSC6: 19.9–20.9 Mb) was co-localised for SGE and DGE in this study. The previously mentioned *SLC38A7* located in this QTL window is expressed in GABAergic and other neurons in brain, and in liver and skeletal muscle. In addition to neuropsychiatric traits, this gene has been implicated in energy metabolism and cell growth^44^. Therefore, *SLC38A7* is considered as a potential candidate gene for both SGE and DGE on ADG.

We previously reported the identification of several QTLs for SGE and DGE using a standard GWAS approach^45^. In the previous study, the mean phenotypic value of ADG for unrelated pen partners and the phenotypic value of individual ADG were used as the naïve SGE and DGE for the GWAS, respectively. Therefore, several other fixed and random effects were not previously corrected compared with the current study. However, an extended animal model was used to compute BLUP estimates of SGE and DGE on ADG in this study. Subsequently, the two BLUP estimates were used as dependent variables for the ssGWAS. We found that there is a lack of concordance between our previous and current GWAS results. No overlapping QTL was identified in SSC1 and SSC2. The previous study identified significant SNP markers in SSC6. However, these markers were not co-localised with the QTL identified in the current study. This lack of concordance may not be surprising, because we used two different types of dependent variables for the two different GWAS approaches. Nevertheless, we argue that the current ssGWAS results are more plausible, because the extend BLUP model in this study is likely to be better fit for the social interactions between pigs. Moreover, we identified a list of positional candidate genes involved in neuropsychiatric processes that are more relevant to SGE in this study.

### Biological pathway and network analyses of SGE and DGE on ADG in pigs

#### SGE on ADG

**Figure 2:**
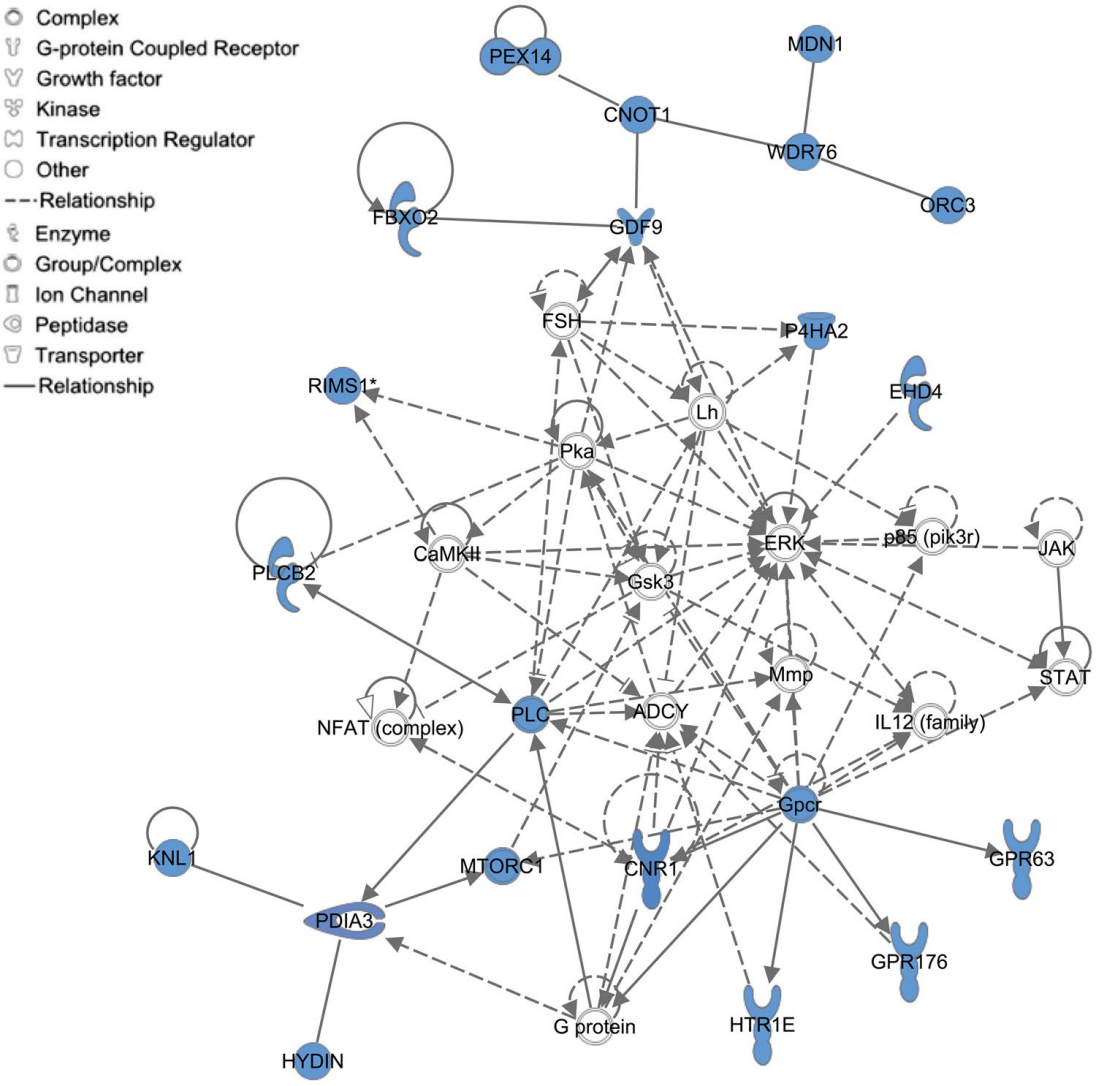
Gene network of interactions between GWAS positional candidate genes using ingenuity pathway analysis (IPA). Inter-relationship among molecules were determined using information stored in the IPA repository. The blue label indicates the positional candidate genes from QTL windows for SGE on ADG.

To evaluate the association of a curated set of genes (i.e., biological pathways) with the SGE on ADG, a total of 199 positional candidate genes, which are located within the QTL windows, were uploaded to the Enrichr database^46^. Functional annotation of the positional candidate genes to biological processes is presented in Table 2. The top three biological pathways significantly enriched in the genes for the SGE on ADG were related to Fc epsilon RI signaling, choline metabolism, and α-linolenic acid metabolism. Furthermore, several genes (*PLA2G4F*, *PLA2G4D*, *PLA2G4E*, *PLA2G4B*, and *PLCB2*) were overrepresented in both long-term depression and serotonergic synapse categories. Another neuropsychiatric function for SGE on ADG were retrograde endocannabinoid signaling. Five genes (*RIMS1*, *GABRR1*, *GABRR2*, *CNR1*, and *PLCB2*) were overrepresented in this pathway. As a complementary method to biological pathway analysis, a network-based analysis was conducted to establish gene network associated with the SGE on ADG using Ingenuity Pathway Analysis (IPA)^47^. The IPA identified a gene–gene interaction network with a score of 31. This network contained 18 of the positional candidate genes detected by ssGWAS (Fig. 2). Among these 18 genes, four (*CNOT1*, *HYDIN*, *RIMS1*, and *PLCB2*) of them were listed in the SZDB, which is a database for schizophrenia genetic research^48^. Moreover, the IPA revealed that this network is associated with cell morphology, developmental disorder, and nervous system development and function.

**Table 2:** Top significant pathways for the positional candidate genes located within the QTL windows for SGE on ADG in Landrace pigs.

Trait	Pathway name	*P*-value*	Genes
SGE	Fc epsilon RI signaling pathway	< 0.01	*PLA2G4F, PLA2G4D, PLA2G4E, PLA2G4B, IL4, IL5, IL13*
SGE	Choline metabolism in cancer	< 0.01	*PLA2G4F, PLA2G4D, PLA2G4E, PLA2G4B, SLC22A4, SLC22A5, MTOR*
SGE	alpha-Linolenic acid metabolism	< 0.01	*PLA2G4F, PLA2G4D, PLA2G4E, PLA2G4B*
SGE	Long-term depression	< 0.01	*PLA2G4F, PLA2G4D, PLA2G4E, PLA2G4B, PLCB2*
SGE	Serotonergic synapse	< 0.01	*PLA2G4F, PLA2G4D, PLA2G4E, PLA2G4B, PLCB2, HTR1E*
SGE	Phenylalanine, tyrosine and tryptophan biosynthesis	< 0.01	*TAT, GOT2*
SGE	Arginine and proline metabolism	< 0.01	*P4HA2, CKMT1A, GOT2, SRM*
SGE	Asthma	< 0.01	*IL4, IL5, IL13*
SGE	Phospholipase D signaling pathway	< 0.01	*PLA2G4F, PLA2G4D, PLA2G4E, PLA2G4B, PLCB2, MTOR*
SGE	Ubiquinone and other terpenoid-quinone biosynthesis	< 0.01	*COQ3, TAT*
SGE	Cysteine and methionine metabolism	< 0.05	*TAT,GOT2, SRM*
SGE	IL-17 signaling pathway	< 0.05	*IL4, IL5, IL13, MAP3K7*
SGE	Retrograde endocannabinoid signaling	< 0.05	*RIMS1, GABRR2, GABRR1, CNR1, PLCB2*
SGE	T cell receptor signaling pathway	< 0.05	*IL4, IL5, PAK6, MAP3K7*
SGE	Protein processing in endoplasmic reticulum	< 0.05	*PDIA3, FBXO2, FBXO6, EIF2AK4, UBE2J1*
SGE	Autophagy	< 0.05	*RRAGD, EIF2AK4, MAP3K7, MTOR*

*Nominal *P*-value.

#### DGE on ADG

**Figure 3:**
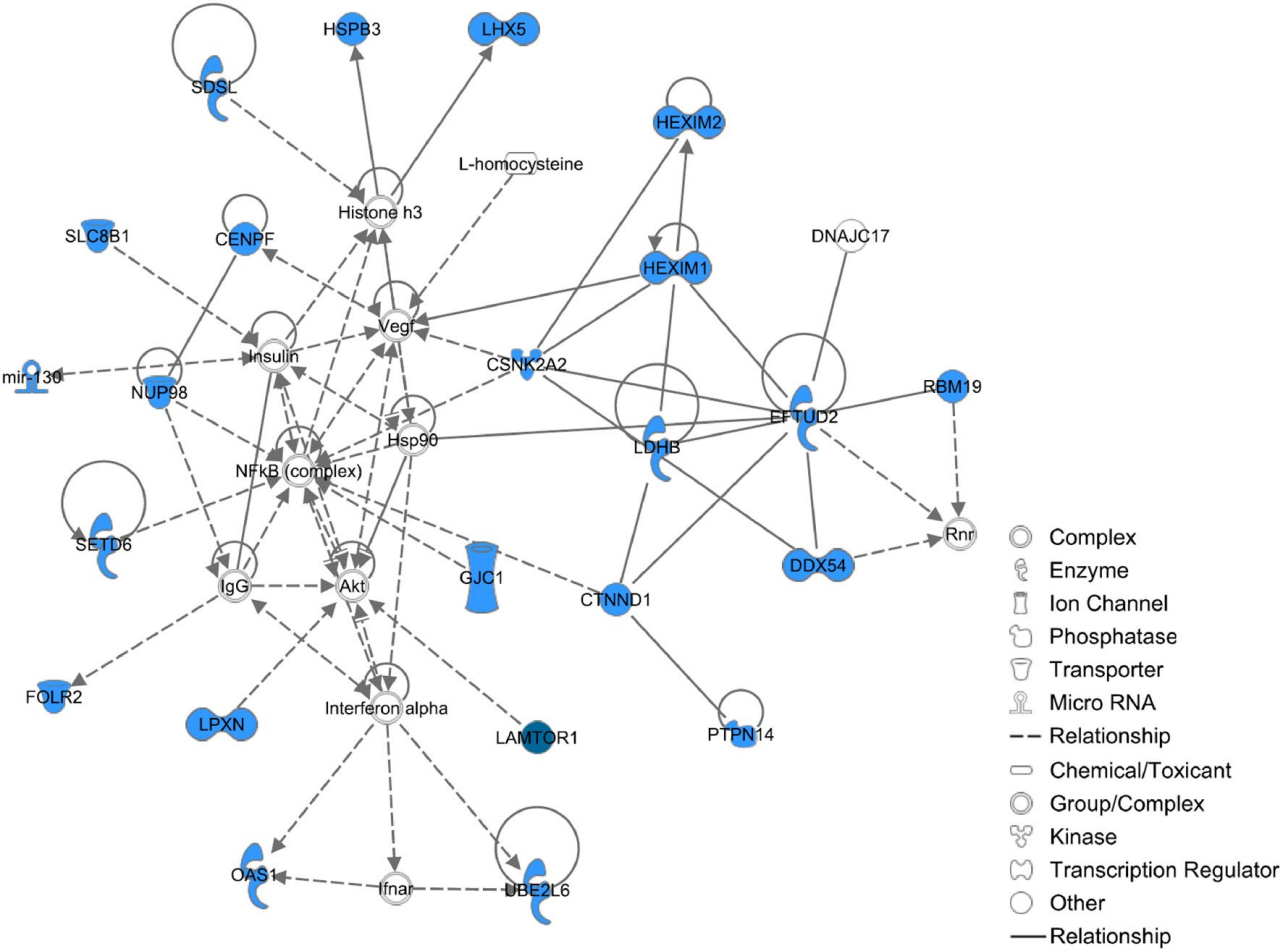
Gene network of interactions between GWAS positional candidate genes using ingenuity pathway analysis (IPA). Inter-relationship among molecules were determined using information stored in the IPA repository. The blue label indicates the positional candidate genes from QTL windows for DGE on ADG.

To conduct biological pathway analyses for the DGE on ADG, a total of 108 positional candidate genes were used (Table 3). The top biological pathway for the DGE on ADG was related to cysteine and methionine metabolism. Four genes (*LDHB*, *SDS*, *SDSL*, and *GOT2*) involved in this amino acid metabolism were overrepresented in this pathway. Castellano et al. reported that cysteine and methionine levels can affect preadipocytes proliferation and differentiation which can be relevant to fatness traits in pigs^49^. The Enricher database identified a signaling pathway that regulates pluripotency of stem cells for the DGE on ADG. Three genes (*WNT9B, LHX5*, and *WNT3*) were overrepresented in this signaling pathway. It is well documented that WNT protein-mediated signaling plays an important role in the pathogenesis of obesity^50,51^. Additionally, the mTOR signaling pathway was also detected by this analysis; this pathway included three genes (*WNT9B, LAMTOR1*, and *WNT3*). The mTOR pathway orchestrates multiple major cellular processes, such as cell growth^52,53^. The IPA detected a gene–gene interaction network with score of 49. This network contained 23 of the positional candidate genes detected by ssGWAS for DGE on ADG (Fig. 3). The IPA elucidated that the functions of this network included cardiovascular system development and function, cellular assembly and organisation as well as cellular function and maintenance.

**Table 3:** Top pathways for the positional candidate genes located within the QTL windows for DGE on ADG in Landrace pigs.

Trait	Pathway name	*P*-value*	Genes
DGE	Cysteine and methionine metabolism	< 0.01	*LDHB, SDS, SDSL, GOT2*
DGE	Aldosterone-regulated sodium reabsorption	< 0.05	*SCNN1G, SCNN1B*
DGE	Measles	< 0.05	*OAS1, OAS2, CSNK2A2*
DGE	Signaling pathways regulating pluripotency of stem cells	< 0.05	*WNT9B, LHX5, WNT3*
DGE	mTOR signaling pathway	< 0.05	*WNT9B, LAMTOR1, WNT4*

*Nominal *P*-value.

## Conclusion

Using an extended BLUP model, we found that genetic variation in pen mates (i.e., SGE) influences the variation in ADG in Landrace pigs. We also provide a list of QTLs and positional candidates associated with SGE and DGE on ADG using ssGWAS. The identified positional candidate genes for SGE on ADG (*PRDM13*, *MAP3K7*, *CNR1*, *HTR1E*, *IL4*, *IL5*, *IL13*, *KIF3A*, *EFHD2*, *SLC38A7*, *mTOR*, *CNOT1*, *PLCB2*, *GABRR1*, and *GABRR2*) have biological roles that are strongly associated with neuropsychiatric processes. Furthermore, the post-GWAS pathway and network analyses also supported the association of neuropsychiatric processes with SGE on ADG. This study contributes to our understanding of the molecular basis of SGE.

## Materials and methods

### Animals and phenotype data

The total numbers of animals in the pedigree of Landrace pigs were 23,152. These pigs were born and raised in a closed nucleus (breeding) farm in the Republic of Korea. Both parents were known for a total of 22,983 individuals. The average inbreeding coefficient was 0.064. The range of inbreeding coefficients was 0.00009–0.304. The observed average family size was 4.12 with ranges of 2–23. The population structures of these breeds were determined using the CFC v1.0 software^54^.

At the same closed nucleus farm, the phenotypic dataset for ADG was obtained by performance tests of Landrace (N = 21,554) pigs between years 2001 and 2015. A total of 4 − 13 pigs of the same sex were placed in each pen to form the groups of pigs. The average group size was 6.8 ± 1.9. As Hong et al. described, the performance evaluations of ADG of pigs started soon after each animal reached a live body weight of 30 kg, and continued until a target weight of 90 kg was attained; pigs were fed ad libitum, and water was constantly accessible through nipple drinkers^55^. On average, fewer than 160 days were required to attain this target weight. The mean and standard deviation of ADG were 804 ± 91 g/day.

### Genotype data

The genomic DNA of pigs was extracted from blood samples collected from jugular veins using a standard protocol. A total of 1,041 Landrace pigs were genotyped using the Illumina PorcineSNP60 v2 BeadChip panel, which included 61,565 SNP markers^56^. This population was previously used in GWAS for the naïve SGE^45^. The quality control process of the genotype data included the removal of individuals with pedigree errors, omission of monomorphic SNP genotypes, SNPs on sex chromosomes or SNPs with minor allele frequencies (< 0.95), genotype call rate of < 0.90, animal missing rate of > 0.90, Hardy–Weinberg equilibrium of 0.15, and the SNPs with displaced segregation distortion^57,58^. After quality control, the final dataset included genotypes from a total of 1,029 pigs. The total number of autosomal SNPs was reduced to 39,136 after 36.4% of SNPs from the original Illumina marker panel were removed.

### Quantitative genetic analysis of SGE

The ADG trait was analysed by the extended animal model below. Bayesian inference using Gibbs sampling procedure was used to estimate (co)variance components of the studied trait. The effects of birth year-month (168 levels), sex (male or female), and group size (10 levels) were fitted as fixed effects. In the model, age at target weight was fitted as a covariate. The models also included the random effects of the physical pen (112 levels), group identity (3,635 levels), litter of birth (6,098 levels), and permanent environment of the mother (1,888 levels). Animals were fitted as a random effect in the model. Canario et al*.* accounted for early-life environmental effects in the SGE model to avoid bias in the estimated genetic parameters for social effects^7^. The following animal model, which includes social genetic and early-life environmental effects was described as Model 7 of Canario et al*.*^7^:

$${\mathbf{y}} \, = \, {\mathbf{Xb}} \, + \, {\mathbf{Z}}_{{\mathbf{D}}} {\mathbf{a}}_{{\mathbf{D}}} + \, {\mathbf{Z}}_{{\mathbf{S}}} {\mathbf{a}}_{{\mathbf{S}}} + \, {\mathbf{Wc}} \, + \, {\mathbf{Vg}} \, + \, {\mathbf{Tpe}} \, + \, {\mathbf{Ul}} \, + \, {\mathbf{Qk}} \, + \, {\mathbf{e}},$$

where **y** is the vector of observations (ADG), **b** is the vector of the fixed effects, **a**_**D**_ is the vector of the random additive DGE, **a**_**S**_ is the vector of the random additive SGE, **c** is the vector for the random pen with ${\mathbf{c}}{ }\sim {\text{N}}\left( {0,{\mathbf{I}}\sigma_{c}^{2} } \right)$, **g** is the vector of the random group with ${\mathbf{g}}{ }\sim {\text{N}}\left( {0,{\mathbf{I}}\sigma_{g}^{2} } \right)$, **pe** is the vector for the random nongenetic permanent effects of the mother with ${\mathbf{pe}} \sim {\text{N}}\left( {0,{\mathbf{I}}\sigma_{pe}^{2} } \right)$, **l** is the vector for the random birth litter, **k** is the vector for the random early-life environment (birth litter of its group mates), and **e** is the vector of the residuals with ${\mathbf{e}} \sim {\text{N}}\left( {0,{\mathbf{I}}\sigma_{e}^{2} } \right)$. **X**, **Z**_**D**_, **Z**_**S**_, **W**, **V**, **U**, **T**, and **Q** are the corresponding incidence matrices. **I** is an identity matrix of appropriate dimensions. To take into account differences in group size, as suggested by Canario et al.^7^, an additional covariate term known as dilution $\left( {\frac{{\text{Average}\;{\kern 1pt} \text{group}\;\text{size}\; - \text{1}}}{{\text{Group}\;\text{size}\; - \text{1}}}} \right)$ was added to the SGE and early-life environmental effects.

DGE and SGE had the following multivariate normal (MVN) distribution:

$$\left[ {\begin{array}{*{20}c} {a_{D} } \\ {a_{S} } \\ \end{array} } \right]$$ ~ MVN (0, **C** ⊗ **A**), in which **C** is defined by the matrix $$\left[ {\begin{array}{*{20}c} {\sigma_{{a_{D} }}^{2} } & {\sigma_{{a_{D} a_{S } }} } \\ {\sigma_{{a_{D } a_{S } }} } & {\sigma_{{a_{S} }}^{2} } \\ \end{array} } \right]$$, $\sigma_{{a_{S} }}^{2}$ is the variance of social genetic effects, $\sigma_{{a_{D} a_{S } }}$ is the covariance between DGE and SGE, and **C** ⊗ **H** denotes the Kronecker product of two matrices. The relationship matrix **H**, in single-step evaluation, defines the relationship among genotyped and non-genotyped animals. The inverse of the **H** matrix is rather simple in structure^59,60^ and can be given as:

$${\mathbf{H}}^{{ - \text{1}}} = \,{\mathbf{A}}^{{ - \text{1}}} + \,\left[ {\begin{array}{*{20}c} {\mathbf{0}} & {\mathbf{0}} \\ {\mathbf{0}} & {{\mathbf{G}}^{{ - \text{1}}} - {\mathbf{A}}_{{\text{22}}}^{{ - \text{1}}} } \\ \end{array} } \right],$$

where ${\mathbf{A}}_{{\text{22}}}$ is the matrix for only genotyped animals (a submatrix derived from the pedigree-based relationship matrix, **A** and **G** is the relationship matrix among individuals based on genomic information. In addition, birth litter and early-life environmental effects had the following multivariate normal (MVN) distribution:

$$\left[ {\begin{array}{*{20}c} l \\ k \\ \end{array} } \right]$$ ~ **MVN** $$\left( {\begin{array}{*{20}c} 0 \\ 0 \\ \end{array} { }, \left[ {\begin{array}{*{20}c} {\sigma_{l}^{2} } & {\sigma_{lk} } \\ {\sigma_{lk} } & {\sigma_{k}^{2} } \\ \end{array} } \right] \otimes { }{\mathbf{I}}} \right)$$, in which $\sigma_{l}^{2}$ is the variance of birth litter effects, $\sigma_{kl}$ is the covariance between birth litter and early-life environment effects, and $\sigma_{k}$ is the variance of early-life environment effects.

To fit a SGE model, GIBBS2F90 with Bayesian inference using Gibbs sampling was used^61^, and the Gibbs samplers were run as single chains 550,000 rounds. The first 50,000 rounds were discarded as burn-in with thinning every 50 samples. This resulted in a total of 10,000 samples used for post-Gibbs analyses, which were completed using POSTGIBBSF90^61^.

According to Bijma^62^, for traits affected by heritable social effects, the variance of total breeding value (TBV) represents the total heritable variation that is exploitable for selection. The TBV of the *i*th animal was defined as follows:

$$TBV_{i} = a_{D,i} + \left( {n - 1} \right)a_{S,i}$$

where n indicates the average size of social groups. The TBV is the heritable effect of an individual on trait values in the population, which is the sum of its DGE ($a_{D,i}$) on its own phenotype and its SGE ($a_{S,i}$) on the phenotypes of its *n* − 1 group mates. Bijma (2011) also stated that the total heritable variance determines the population’s potential in response to selection and can be expressed as^62^:

$$\sigma_{TBV}^{2} = \sigma_{{a_{D} }}^{2} + 2\left( {n - 1} \right)\sigma_{{a_{D} a_{S} }} + \left( {n - 1} \right)^{2} \sigma_{{a_{S} }}^{2}$$

According to Canario et al., the phenotypic variance for such a model can be calculated as follows^7^:

$$\sigma_{P}^{2} = { }\sigma_{{a_{D} }}^{2} { } + { }\left( {n{ }{-}{ }1} \right)\sigma_{{a_{S} }}^{2} { } + { }\sigma_{c}^{2} { } + { }\sigma_{g}^{2} { } + { }\sigma_{pe}^{2} { } + { }\sigma_{l}^{2} { } + { }\left( {n{ }{-}{ }1} \right)\sigma_{k}^{2} { } + { }\sigma_{e}^{2}$$

The total heritable variance can be expressed relative to phenotypic variance (Bergma et al. 2008) as follows:

$$T^{2} = \frac{{\sigma_{TBV}^{2} }}{{\sigma_{P}^{2} }}$$

### ssGWAS

A ssGWAS, which jointly uses genotype, phenotype, and pedigree information in one step, was conducted on the vector of random additive SGE (i.e., **a**_**S**_). In this ssGWAS approach, greater power and more precise estimates of variance components can be achieved by including non-genotyped animals if the number of genotyped animals is limited^63^. The **G** matrix was constructed with method 1 in VanRaden (2008)^64^:

$${\mathbf{G}} \, = \, {\mathbf{ZDZ^{\prime}}}q$$

where **Z** is a matrix of gene content that contains genotype adjusted for allele frequencies; **D** is a diagonal matrix of weights for variances of SNPs (initially **D** = **I**); and *q* is a weighting factor. This factor can be derived by ensuring that the average diagonal in **G** is close to that of **A**_22_^65^. The SNP effects and weights were derived as follows:

1. Let D = I in the first step.
2. Calculate breeding values
3. Convert breeding values to SNP effects ${\hat{\mathbf{u}}}$ = ${\mathbf{DZ^{\prime}}}\left[ {{\mathbf{ZDZ^{\prime}}}} \right]^{ - 1} {\hat{\mathbf{a}}}_{{\varvec{g}}}$, where ${\hat{\mathbf{a}}}_{{\varvec{g}}}$ is the breeding values of the animals which were also genotyped.
4. Calculate the weight for each SNP: $d_{i}$ = ${\hat{\text{u}}}^{2} 2p_{i} \left( {1 - p_{i} } \right)$, where i is the i-th SNP
5. Normalise SNP weight to retain the total genetic variance constant.

The breeding values, the D matrix and the SNP effects were iteratively recalculated over two iterations, as suggested by Wang et al.^63^.

The percentage of additive genetic variance explained by *i*-th region was computed as below:

$$\frac{{Var \left( {a_{i} } \right)}}{{\sigma_{a}^{2} }} \times 100 = \frac{{Var \left( {\mathop \sum \nolimits_{j = 1}^{10} Z_{j} {\hat{\mathbf{u}}}_{j} } \right)}}{{\sigma_{a}^{2} }}{ } \times 100$$

where $a_{i}$ is genetic value of the *i*-th region that includes uninterrupted 10 adjacent SNPs, $\sigma_{a}^{2}$, the additive genetic variance; $Z_{j}$, the vector of gene content of the *j*-th SNP for all individuals; and ${\hat{\mathbf{u}}}_{j}$, marker effect of the *i*-th SNP within the *i*-th region. The fraction of additive genetic variance explained by SNPs within non-overlapping consecutive 1-Mb segments was evaluated. The QTL windows were identified and located in the pig genome using the database available in the NCBI *Sus scrofa* Build 11.1.

### Search for positional candidate genes

The windows (chromosomal segments) that accounted for equal to or greater than 0.5% of the additive genetic variance from GWAS were determined to be the QTL regions^66–68^. The QTL windows were identified and located for candidate genes using the *Sus scrofa* Build 11.1 assembly and tools available in NCBI.

### Biological pathway and network analyses

The selected candidate genes were uploaded into the Enricher database to investigate relevant biological pathways^46^. The gene interaction network analysis was conducted using IPA tools as a complementary analysis to the Enricher biological pathway analysis^47^. The same list of candidate genes used for the pathway analysis were uploaded into the IPA for network analysis. These analyses were conducted with their default settings.

### Ethical approval

All experimental protocols were approved by the Institutional Animal Care and Use Committee (IACUC) at the National Institute of Animal Science (NIAS), Republic of Korea. All methods in this study were carried out in accordance with relevant guidelines and regulations. Necessary approval was obtained from the IACUC of the NIAS, Republic of Korea (Approval number: NIAS20191709).

## Supplementary information

Supplementary Information.
