# Supplementary material for: Single-step genome-wide association study for social genetic effects and direct genetic effects on growth in Landrace pigs
Source: Sci Rep. 2020 Sep 11;10:14958. doi: 10.1038/s41598-020-71647-x (PMC7486944; doi:10.1038/s41598-020-71647-x)
Supplement: Supplementary file 1 — Supplementary Information. [file 41598_2020_71647_MOESM1_ESM.docx]

**Supplementary table 1**. QTLs associated with SGE on ADG in Landrace pigs

| **QTL window**  **(Chr:BP range)^a^** | | **% Variance^b^** | **Genes^c^** |
| --- | --- | --- | --- |
| 1:61,323,966-62,318,714 | 5.36 | | *-* |
| 1:66,491,115-67,455,613 | 4.96 | | *CCNC, PRDM13, MCHR2, SIM1, ASCC3* |
| 1:58,013,620-59,007,636 | 4.90 | | *BACH2, MAP3K7* |
| 1:63,704,733-64,674,794 | 3.59 | | *FHL5, GPR63, NDUFAF4, KLHL32, MMS22L* |
| 1:55,627,922-56,615,016 | 3.52 | | *ZNF292, GJB7, SMIM8, C1H6orf163, CFAP206, SLC35A1, RARS2, ORC3, AKIRIN2, TRNAE-UUC, SPACA1, CNR1* |
| 1:62,534,115-63,529,967 | 2.36 | | *MANEA* |
| 1:130,611,566-131,488,278 | 2.19 | | *RAD51, KNL1, TRNAS-GCU, RPUSD2, C1H15orf57, CHST14, BAHD1, IVD, KNSTRN, DISP2, C1H15orf52, INAFM2, PLCB2, ANKRD63, PAK6, BUB1B, BMF, SRP14, EIF2AK4, GPR176* |
| 1:59,091,108-60,027,416 | 2.14 | | *-* |
| 1:65,337,586-66,322,048 | 1.97 | | *POU3F2, FBXL4, FAXC, COQ3* |
| 1:60,304,507-61,303,912 | 1.88 | | *EPHA7* |
| 1:127,417,689-128,323,543 | 1.84 | | *WDR76, MFAP1, SERINC4, SERF2, ELL3, PDIA3, CATSPER2, STRC, CKMT1A, PPIP5K1, MAP1A, MIR2366-1, TP53BP1, TUBGCP4, ZSCAN29, ADAL, LCMT2, TGM7, TGM5, EPB42, CCNDBP1, TMEM62, MIR9838, UBR1* |
| 1:129,025,960-129,994,936 | 1.34 | | *GANC, TMEM87A, VPS39, PLA2G4F, PLA2G4D, PLA2G4E, EHD4, SPTBN5, PLA2G4B, JMJD7, MAPKBP1, MGA, TYRO3, RPAP1, LTK, ITPKA, RTF1, NDUFAF1, NUSAP1* |
| 1:56,925,724-57,920,577 | 1.21 | | *RNGTT, PNRC1, SRSF12, PM20D2, GABRR1, GABRR2, UBE2J1, RRAGD, ANKRD6, LYRM2, MDN1, CASP8AP2, GJA10, BACH2* |
| 1:54,422,838-55,422,120 | 1.09 | | *NT5E, SNX14, SYNCRIP, HTR1E* |
| 1:53,352,202-54,338,731 | 0.79 | | *CEP162, TBX18* |
| 1:111,455,138-112,433,789 | 0.60 | | *ICE2, ANXA2, SNRPE, FOXB1, BNIP2, GTF2A2, GCNT3* |
| 1:51,555,713-52,528,119 | 0.58 | | *RIMS1, KCNQ5* |
| 2:134,496,051-135,476,573 | 0.55 | | *P4HA2, PDLIM4, SLC22A4, SLC22A5, IRF1, IL5, RAD50, KIF3A, IL13, IL4, CCNI2, SEPT8, SOWAHA, SHROOM1, GDF9, LEAP2, AFF4, ZCCHC10, HSPA4* |
| 6:73,729,318-74,701,809 | 0.95 | | *KAZN, TMEM51, FHAD1, EFHD2, CTRC, CELA2A* |
| 6:75,960,523-76,930,395 | 0.92 | | *PADI4, PADI6, RCC2, ARHGEF10L, ACTL8, IGSF21* |
| 6:19,926,572-20,905,171 | 0.87 | | *CFAP20, CSNK2A2, CCDC113, PRSS54, GINS3, NDRG4, SETD6, CNOT1, SLC38A7, GOT2* |
| 6:70,841,466-71,826,762 | 0.59 | | *PEX14, CASZ1, MIR9861, TARDBP, MASP2, SRM, EXOSC10, MTOR, ANGPTL7, UBIAD1, DISP3, FBXO2, FBXO44, FBXO6, MAD2L2, DRAXIN, AGTRAP* |
| 6:14,164,821-15,036,822 | 0.59 | | *HYDIN, CMTR2, CALB2, TRNAM-CAU, ZNF19, ZNF23, CHST4, TAT, MARVELD3, PHLPP2, AP1G1, ATXN1L, ZNF821, IST1, DHODH, HP, TXNL4B, DHX38, PMFBP1* |

^a^Chr, Chromosome; BP range, Range of significant chromosome region in base pair position

^b^% Variance, Percent variance explained by QTL window

^c^Genes, Annotated genes located within the QTL window

**Supplementary table 2**. QTLs associated with DGE on ADG in Landrace pigs

| **QTL window**  **(Chr:BP range)^a^** | **% Variance^b^** | **Genes^c^** |
| --- | --- | --- |
| 2:12451845-13371349 | 0.61 | *CNTF, ZFP91, LPXN, TRNAV-UAC, LDHB, CTNND1, BTBD18, SELENOH, TMX2, MED19, ZDHHC5, CLP1, YPEL4, MIR130A, SERPING1, TRNAM-CAU, UBE2L6, SMTNL1* |
| 3:22771925-23755924 | 0.74 | *COG7, SCNN1B, SCNN1G, USP31, HS3ST2, OTOA, METTL9, IGSF6* |
| 6:19903679-20886479 | 0.56 | *MMP15, CFAP20, CSNK2A2, CCDC113, PRSS54, GINS3, NDRG4, SETD6, CNOT1, SLC38A7, GOT2* |
| 9:128233367-129225630 | 1.10 | *KCNK2, CENPF, PTPN14* |
| 9:6362659-7355808 | 0.75 | *NUP98, CHRNA10, ART1, ART5, RNF121, IL18BP, NUMA1, LRRC51, LAMTOR1, ANAPC15, FOLR1, FOLR2, INPPL1, PHOX2A, CLPB, PDE2A, MIR139, ARAP1, STARD10, ATG16L2* |
| 10:58686370-59656315 | 0.64 | *CAMK1D* |
| 12:17674657-18598973 | 0.69 | *WNT3, WNT9B, GOSR2, RPRML, LYZL6, PLEKHM1, ARHGAP27, MAP3K14, SPATA32, FMNL1, HEXIM2, HEXIM1, ACBD4, PLCD3, NMT1, DCAKD, C1QL1, KIF18B, GFAP, CCDC103, EFTUD2, HIGD1B, GJC1, ADAM11* |
| 14:37930238-38927125 | 0.75 | *RBM19, LHX5, SDSL, SDS, PLBD2, DTX1, RASAL1, CFAP73, DDX54, RITA1, IQCD, TPCN1, SLC8B1, OAS2, OAS1, RPH3A* |
| 14:135163946-136146067 | 0.66 | *DHX32, FANK1, ADAM12* |
| 16:33,008,580-34002361 | 1.05 | *ARL15, HSPB3, SNX18* |
| 17:53492314-54480182 | 0.58 | *TSHZ2* |

^a^Chr, Chromosome; BP range, Range of significant chromosome region in base pair position

^b^% Variance, Percent variance explained by QTL window

^c^Genes, Annotated genes located within the QTL window
